# Oral microbial profile variation during canine ligature-induced peri-implantitis development

**DOI:** 10.1186/s12866-020-01982-6

**Published:** 2020-09-29

**Authors:** Shichong Qiao, Dongle Wu, Mengge Wang, Shujiao Qian, Yu Zhu, Junyu Shi, Yongjun Wei, Hongchang Lai

**Affiliations:** 1 Department of Implant Dentistry, Shanghai Ninth People’s Hospital, College of Stomatology, Shanghai Jiao Tong University School of Medicine, National Clinical Research Center for Oral Diseases, Shanghai Key Laboratory of Stomatology & Shanghai Research Institute of Stomatology, Shanghai, 200011 PR China; 2 Key Laboratory of Advanced Drug Preparation Technologies, Ministry of Education & School of Pharmaceutical Sciences, Zhengzhou University, Zhengzhou, 450001 Henan Province PR China

**Keywords:** Microbiota, Ligature-induced, Canine peri-implantitis, Microbial variation, Keystone taxonomy

## Abstract

**Background:**

Dental implants have become well-established in oral rehabilitation for fully or partially edentulous patients. However, peri-implantitis often leads to the failure of dental implants. The aim of this study was to understand the core microbiome associated with peri-implantitis and evaluate potential peri-implantitis pathogens based on canine peri-implantitis model.

**Results:**

In this study, three beagle dogs were used to build peri-implantitis models with ligature-induced strategy. The peri-implant sulcular fluids were collected at four different phases based on disease severity during the peri-implantitis development. Microbial compositions during peri-implantitis development were monitored and evaluated. The microbes were presented with operational taxonomic unit (OTU) classified at 97% identity of the high-throughput 16S rRNA gene fragments. Microbial diversity and richness varied during peri-implantitis. At the phylum-level, *Firmicutes* decreased and *Bacteroides* increased during peri-implantitis development. At the genus-level, *Peptostreptococcus* decreased and *Porphyromonas* increased, suggesting peri-implantitis pathogens might be assigned to these two genera. Further species-level and co-occurrence network analyses identified several potential keystone species during peri-implantitis development, and some OTUs were potential peri-implantitis pathogens.

**Conclusion:**

In summary, canine peri-implantitis models help to identify several potential keystone peri-implantitis associated species. The canine model can give insight into human peri-implantitis associated microbiota.

## Background

With the development of oral technologies, dental implants are a highly successful and predictable treatment for replacing missing teeth, which can rehabilitate oral health-related quality of life [1]. However, breakdown of soft and hard tissue around osseointegrated implants would result in the failure of dental implants known as inflammatory peri-implant disease [2–4]. Clinically, inflammatory peri-implant diseases are categorized into peri-implant mucositis and peri-implantitis [5, 6]. Peri-implant mucositis is defined as a reversible inflammatory reaction in the soft tissues surrounding a functioning implant, while peri-implantitis is described as inflammatory reactions involving supporting bone loss [5, 6]. It has been estimated that the rate of implant-based and subject-based peri-implantitis are 9.25% and 19.83%, respectively [7]. In another study, the prevalence of peri-implantitis is up to 22% [8]. Therefore, understanding the mechanism that induces peri-implantitis is essential in current restorative dentistry, and might help to find preventive strategies for peri-implantitis and provide effective peri-implantitis treatment methods [9, 10].

It is generally accepted that peri-implantitis is a complex disease with multiple risk factors, and it initiates from a bacterial challenge and host overreaction [11, 12]. Much effort has been made to study the peri-implant biofilm and its difference from periodontal biofilm [13], and reveal the bacterial colonization in healthy and diseased states [14]. The comparison between diseased peri-implant and healthy periodontal tissues has been applied [15]. Several potential microbial strains, such as *Porphyromonas gingivalis* and *Prevotella intermedius*, have been detected in oral biofilm at the peri-implantitis sites. However, there is no clear consensus for core microbiota associated with peri-implantitis [16]. Traditional cultured strategies provide valuable information regarding known pathogens, but limited data on unculturable species and microbiota during peri-implantitis development are available. Global investigation of microbiota variation during peri-implantitis development would make it possible to identify peri-implantitis associated microbiota [17, 18], and high-throughput sequencing of 16S rRNA gene technology provides a wealth of data pertaining to the differences between healthy and diseased implants [19, 20].

Experimental peri-implantitis animal models, such as mice and dogs, have been developed to study the mechanisms and development of marginal bone loss [8, 21]. In previous animal studies, undisturbed peri-implant plaque accumulation often led to negligible or no marginal bone loss [22]. In order to obtain reliable peri-implantitis animal models, sub-marginal ligatures of cotton, silk or other materials have been used to induce and speed up the peri-implantitis process [19, 23, 24]. Normally, sub-marginal ligation results in significant bone loss in a few weeks, and the peri-implantitis model can be established in a short time [25, 26]. However, investigation of microbial dynamics during peri-implantitis model establishment had not been reported.

Here, the present study was designed to conduct a canine peri-implantitis model using sub-marginal ligatures method. The clinical parameters at different peri-implantitis phases were evaluated, and microbial variation associated with the severity of tissue destruction and inflammatory progression of experimental peri-implantitis were investigated. The core microbiota associated with peri-implantitis and their networks were analyzed, and the potential keystone microbial taxa associated with peri-implantitis were discussed.

## Results

### Establishment of peri-implantitis models

Eight teeth of each dog were successfully removed with minimal trauma (Supplementary Figure 1). After healing and recovery, the peri-implantitis induction was implemented. Overall, healing after tooth extraction and implant placement was uneventful at all surgical sites. Initial stability of each implant was confirmed after implant installation, and no implant loss was observed during the entire experimental period.

**Fig. 1:**
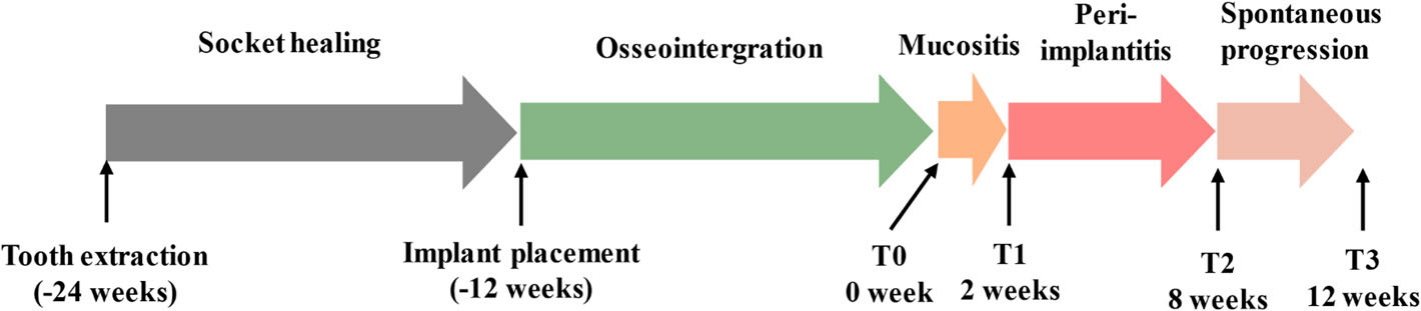
Peri-implantitis development timeline and the sampling time points

No visible inflammatory signs were observed before ligatures were applied at the implant sites (Phase T0) (Fig. 1 and Supplementary Figure 1). As expected, heavy plaque accumulation, bleeding on probing and probing depths increased following ligature placement, and samples at peri-implantitis timepoints were collected (Phase T1 to Phase T3). After each sampling, the implants were kept immobile by manually checking.

**Fig. 2:**
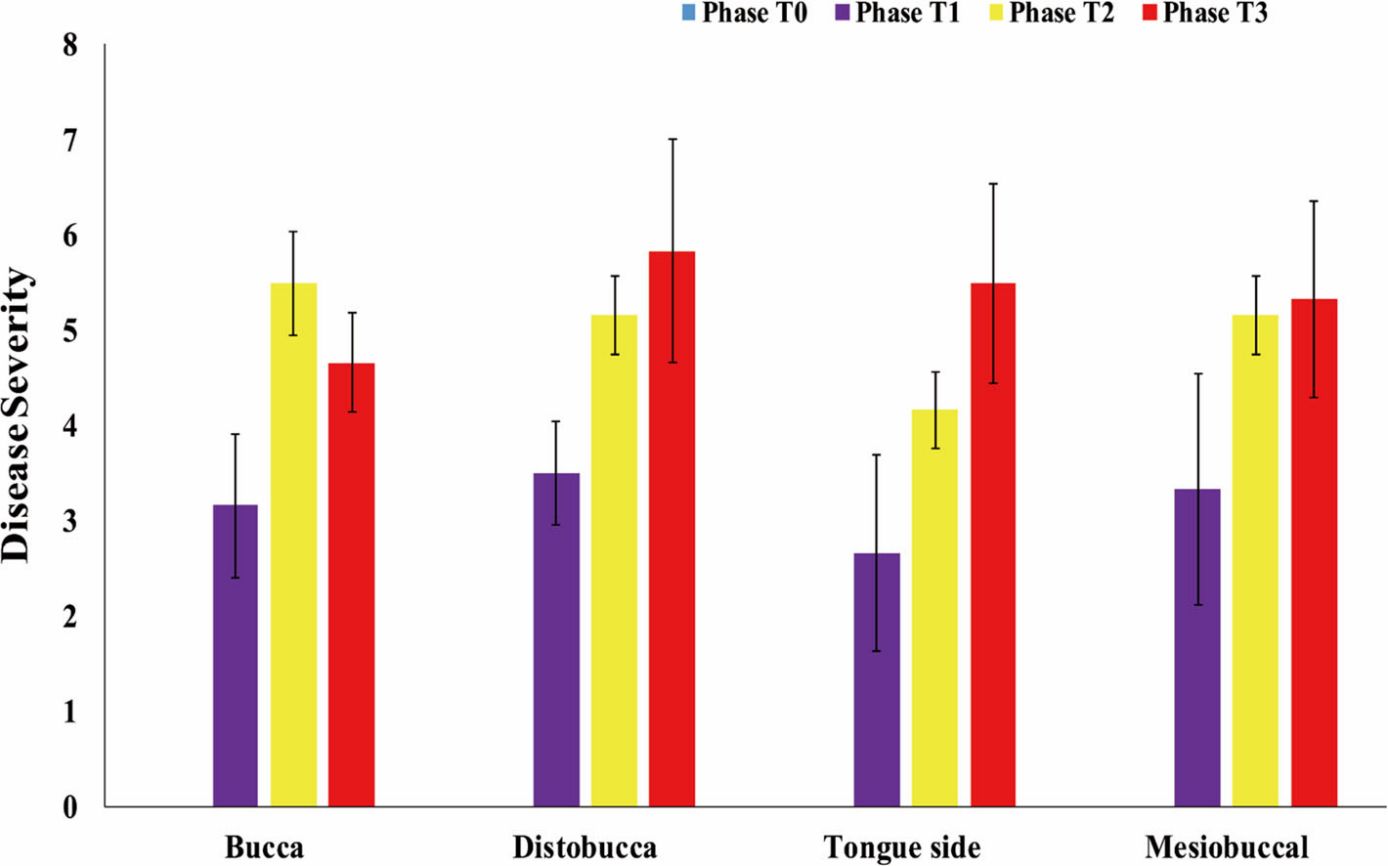
Disease severity of the bucca, distobucca, tongue side and mesiobuccal of the implants during Peri-implantitis development. The error bars represent the standard deviation of six replicates of each site around the implant

The disease severity around the inflammatory teeth were evaluated with heavy depth measurements (Fig. 2). The disease severity around the distobuccal, tongue side, and mesiobuccal increased during the peri-implantitis induction period (Phase T1-T2) and self-spontaneous recovery period (Phase T3). Meanwhile, the disease severity around buccal increased during the peri-implant inflammation period (Phase T1 and Phase T2), and the disease severity alleviated during the spontaneous recovery period (Phase T3). Taken together, teeth health deteriorated after peri-implantitis induction (Phase T1 to Phase T3) (Fig. 2).

### Diversity of the peri-implant sulcus samples at different phases

The 24 peri-implant sulcus samples collected at Phase T0 to T3 were sequenced, and the data were used to indicate microbial diversity of these samples (Supplementary Figure 2). The operational taxonomic unit (OTU) numbers of these 24 samples were 206–445. The average OTU numbers of Phase T0 to T3 were 294, 337, 389, and 305, respectively (Table 1). Only the OTU numbers between Phase T0 and Phase T2, as well as Phase T2 and Phase T3 displayed differences (Supplementary Table 1). The alpha parameters of Shannon_2, simpson, dominance and equitability at Phase T0 were higher than the respective parameters at Phase T1 to T3 (Table 1). Moreover, these four alpha parameters displayed differences or significant differences between the healthy state and the peri-implantitis period, including Phase T0 and Phase T1, Phase T0 and Phase T2, and Phase T0 and Phase T3 (Supplementary Table 1). However, these four alpha parameters displayed no differences between Phase T1 and Phase T2, Phase T1 and Phase T3, and Phase T2 and Phase T3 (Table 1 and Supplementary Table 1).

**Table 1:** Average alpha diversity parameters during Peri-implantitis development of the dog teeth

	Richness	Chao1	Shannon_2	Simpson	Dominance	Equitability
Phase T0	294 ± 78	295 ± 78	4 ± 0.8	0.2 ± 0.1	0.8 ± 0.1	0.5 ± 0.1
Phase T1	337 ± 76	339 ± 75	5.3 ± 0.5	0.07 ± 0.03	0.9 ± 0.03	0.6 ± 0.08
Phase T2	389 ± 34	390 ± 34	5.6 ± 0.4	0.05 ± 0.02	1 ± 0.02	0.7 ± 0.04
Phase T3	305 ± 78	307 ± 76	5.1 ± 0.4	0.06 ± 0.02	0.9 ± 0.02	0.6 ± 0.07

### Microbiota analyses

**Fig. 3:**
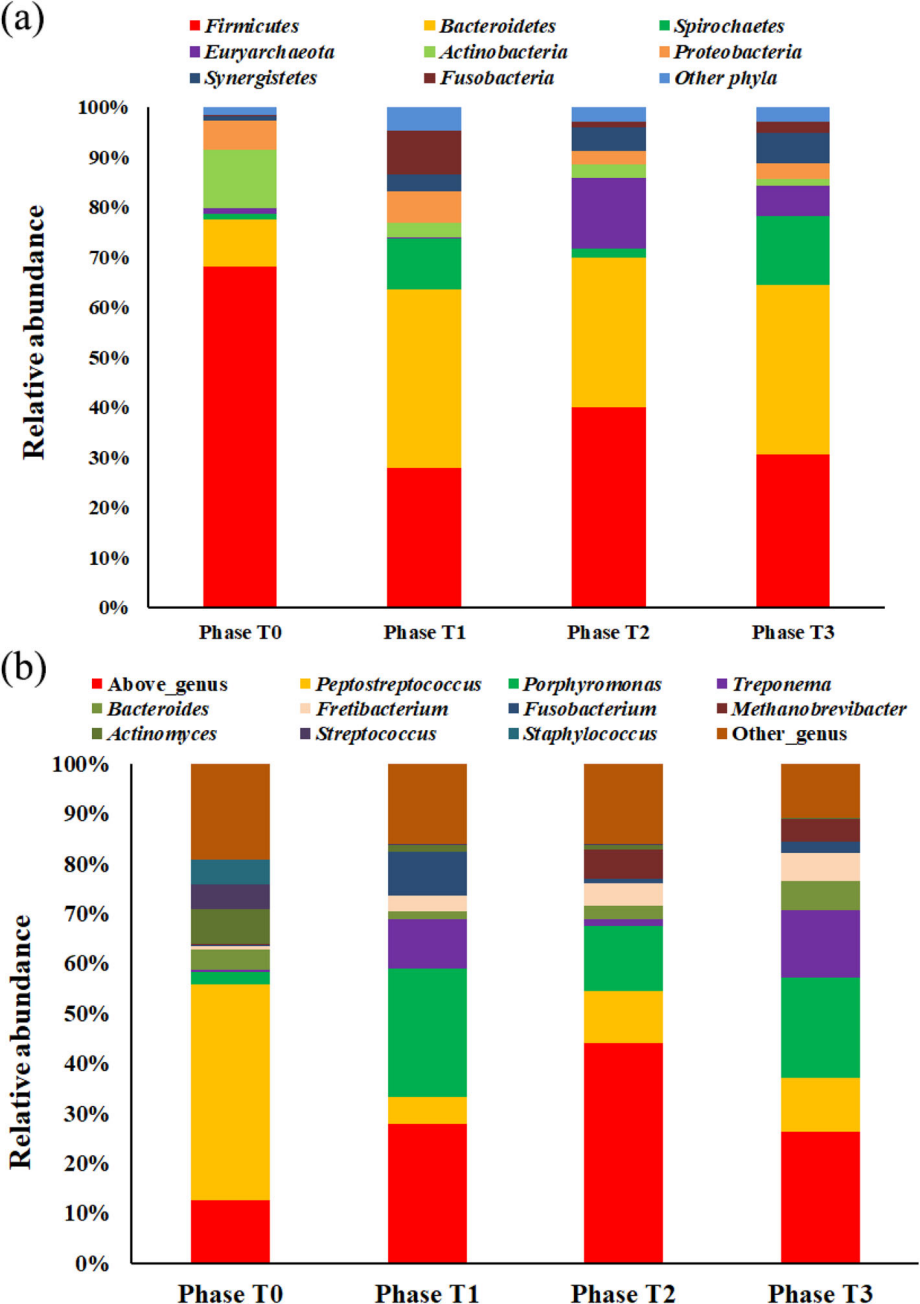
Microbial compositions of the implant samples during Peri-implantitis development at phylum-level (**a**) and genus-level (**b**)

The microbial distribution of the same phases were similar (Supplementary Figure 3), while the microbial distribution of different phases harbored different phyla (Fig. 3a). At Phase T0 of the healthy state, the most dominant phyla were *Firmicutes* (68.1%), *Actinobacteria* (11.7%), and *Bacteroidetes* (9.4%). At Phase T1 during the first 2 weeks of peri-implantitis, the most dominant phyla were *Firmicutes* (27.8%), *Bacteroidetes* (35.8%), and *Spirochaetes* (10.2%). At Phase T2, the most dominant phyla were *Firmicutes* (39.9%), *Bacteroidetes* (30.0%), and *Euryarchaeota* (14.1%). At Phase T3 of the self-spontaneous recovery period, the most dominant phyla were *Firmicutes* (30.7%), *Bacteroidetes* (33.9%), *Spirochaetes* (13.6%), and *Euryarchaeota* (6.1%). The *Firmicutes*, *Bacteroidetes* and *Synergistetes* distribution between Phase T0 and Phase T1 to T3 displayed significant differences. The *Euryarchaeota* distribution between Phase T0 and T2, T0 and T3, T1 and T2, and T1 and T3 displayed significant differences (Supplementary Table 2). Besides, a few other phyla displayed differences between different phases (Supplementary Table 2).

At the genus-level, microbial distribution at different phases changed (Fig. 3b). At Phase T0, the most dominant genera were *Peptostreptococcus* (43.5%), *Actinomyces* (7%), and *Streptococcus* (4.9%). At Phase T1, the most dominant genera were *Portostreptococcus* (25.6%), *Treponema* (10.0%), and *Fusobacterium* (8.8%). At Phase T2, the most dominant genera were *Portostreptococcus* (13.0%), *Peptostreptococcus* (10.5%), and *Methanobrevibacter* (5.7%). At Phase T3, the most dominant genera were *Portostreptococcus* (20.0%), *Treponema* (13.4%), and *Portostreptococcus* (10.9%). Some genera, including *Peptostreptococcus*, *Porphyromonas*, *Treponema* and *Fretibacterium*, displayed significant differences between Phase T0 and Phase T1 to T3. The *Methanobrevibacter* displayed significant differences between T0 and T2, T0 and T3, T1 and T2, and T1 and T3 (Supplementary Table 3). No significant differences of *Bacteroides* and *Streptococcus* between Phase T0 and Phase T1 to Phase T3 were observed.

### The dominant OTUs in the microbiota

Most OTUs with average compositions > 1% in the four phases had > 97% identity with known isolates (Table 2). Besides, OTU distribution at different phases displayed difference or significant difference (Supplementary Table 4). OTU_1, which composes 43.46% at Phase T0, might be one *Peptostreptococcus canis* strain isolated from subgingival plaque from canine oral cavity [27]. Compared with Phase T0, *P*. *canis* composition decreased to 5.5% at Phase T1, 10.5% at Phase T2, and 10.9% at Phase T3. The compositions of OTU_3 which might be *Porphyromonas macacae* increased from < 0.2% at Phase T0 to 6.3% at Phase T1, 1% at Phase T2, and 11% at Phase T3 [28]. OTU_7, which was assigned to the genus of *Porphyromonas*, composed 0.12% at Phase T0, but increased to 0.48% at Phase T1, 5.43% at Phase T2, and 6.71% at Phase T3. OTU_13, which was assigned to the genus of *Fretibacterium*, composed only 0.58% at Phase T0, but it increased to > 3% at Phase T1 to T3. In particular, some methanogens, such as OTU_2 and OTU_4, composed < 0.25% at Phase T0. The composition of OTU_2 and OTU_4 increased to 13.91% at phase T2, and their composition decreased to 6.08% at Phase T3. A few other dominant OTUs were assigned to the typical oral microbiota or clinical pathogens, and the compositions of clinical pathogens increased (Table 2). For example, OTU_19 was assigned to teeth infection associated bacteria *Treponema denticola* [29, 30]. OTU_19 composition at Phase T0 was 0.09%, and its composition increased to 3.37% at Phase T1, 0.79% at Phase T2, and 4.89% at Phase T3.

**Table 2:** The distribution of dominant OTUs identified during Peri-implantitis development and the 16S rRNA gene fragments of their closest isolates and uncultured bacteria

	Closest isolates	Identity	Closest uncultured (nr/nt)	Identity	Phase T0	Phase T1	Phase T2	Phase T3
OTU_1	*Peptostreptococcus canis* (NR_117641.1)	99.50%	Uncultured bacterium (JQ192915.1)	100%	43.46%	5.48%	10.50%	10.89%
OTU_3	*Porphyromonas macacae* (NR_025908.1)	97.88%	Uncultured bacterium (KX437329.1)	100%	0.19%	6.29%	0.95%	10.98%
OTU_7	*Porphyromonas endodontalis* (NR_113085.1)	94.37%	Uncultured *Porphyromonas* sp. canine oral (JN713348.1)	99.30%	0.12%	0.48%	5.43%	6.71%
OTU_13	*Fretibacterium fastidiosum* (NR_108538.1)	95.82%	Uncultured *Fretibacterium sp. Feline* (KM462182.1)	100%	0.58%	3.05%	3.99%	4.66%
OTU_5	*Bacteroides pyogenes* (NR_113048.1)	100%	Uncultured *Bacteroides* (MH755473.1)	100%	3.49%	0.61%	2.32%	5.30%
OTU_9	*Fusobacterium simiae* (NR_113318.1)	99.51	Uncultured *Fusobacterium* sp. canine (JN713401.1)	100%	0.07%	8.22%	0.79%	2.07%
OTU_8	*Porphyromonas gulae* (NR_113088.1)	99.53%	Uncultured bacterium (HM328336.1)	99.76%	0.29%	6.92%	2.65%	0.88%
OTU_4	*Methanobrevibacter oralis* (LN898260.1)	100%	Uncultured *Methanobrevibacter * (JN052095.1)	100%	0.23%	0.09%	5.68%	4.53%
OTU_2	*Methanimicrococcus blatticola* (JQ268014.1)	98.97%	Uncultured *Methanimicrococcus* (LT624863.1)	97.42%	0.24%	0.09%	8.23%	1.55%
OTU_19	*Treponema denticola* (NR_074582.1)	97.91%	Uncutlured *Treponema* (JN713361.1)	100%	0.09%	3.37%	0.79%	4.89%
OTU_10	*Porphyromonas cangingivalis* (NR_113080.1)	100%	Uncultured bacterium (JF241087.1)	100%	0.85%	6.38%	1.72%	0.10%
OTU_6	*Intestinimonas butyriciproduens* (NR_118554.1)	94.1%	Uncultured *Clostridiales* bacterium (JN713380.1)	99.75%	0.40%	0.17%	5.41%	2.56%
OTU_14	*Treponema sp. PT8* (AM980447.1)	100%	Uncultured *Treponema* sp. (GQ424168.1)	99.77%	0.05%	2.75%	0.06%	4.36%
OTU_37	*Odoribacter denticanis strain* (NR_042977.1)	99.76%	*Odoribacter denticanis* canine oral (JN713247.1)	99.76%	0.83%	1.28%	2.29%	1.79%
OTU_16	*Olivibacter sitiensis* (NR_043805.1)	84.15%	Uncultured *Bacteroidia* bacterium (KM462114.1)	100%	0.07%	0.04%	3.87%	1.81%
OTU_17	*Staphylococcus intermedius* NCTC 11048 (NR_113351.1)	100%	Uncultured *Staphylococcus* sp. strain (JX482520.1)	100%	4.94%	0.02%	0.02%	0.07%
OTU_15	*Metaprevotella massiliensis* (NR_147377.1)	90.59%	*Prevotella* sp. canine oral taxon (JN713360.1)	100%	0.09%	0.45%	3.52%	0.91%
OTU_11	*Porphyromonas crevioricanis* (NR_104834.1)	100%	Uncultured bacterium (JF17468.1)	100%	0.04%	2.47%	1.24%	1.16%
OTU_47	*Schaalia canis* (NR_025366.1)	98.34%	Uncultured bacterium (JF223833.1)	100%	3.76%	0.51%	0.35%	0.02%
OTU_26	*Intestinimonas butyriciproducens* (NR_118554.1)	94.84%	Uncultured *Ruminococcaceae* bacterium (MH572181.1)	100%	0.08%	0.94%	1.66%	1.77%
OTU_12	*Labilibacter aurantiacus* (NR_156071.1)	85.31%	Uncultured bacterium (KJ874155.1)	92.47%	0.12%	1.71%	1.40%	1.10%
OTU_30	*Filifactor villosus* (NR_041928.1)	99.01%	*Filifactor* sp. Feline (KM462053.1)	100%	0.19%	2.17%	1.09%	0.59%

### Microbial similarity of the samples at different phases

**Fig. 4:**
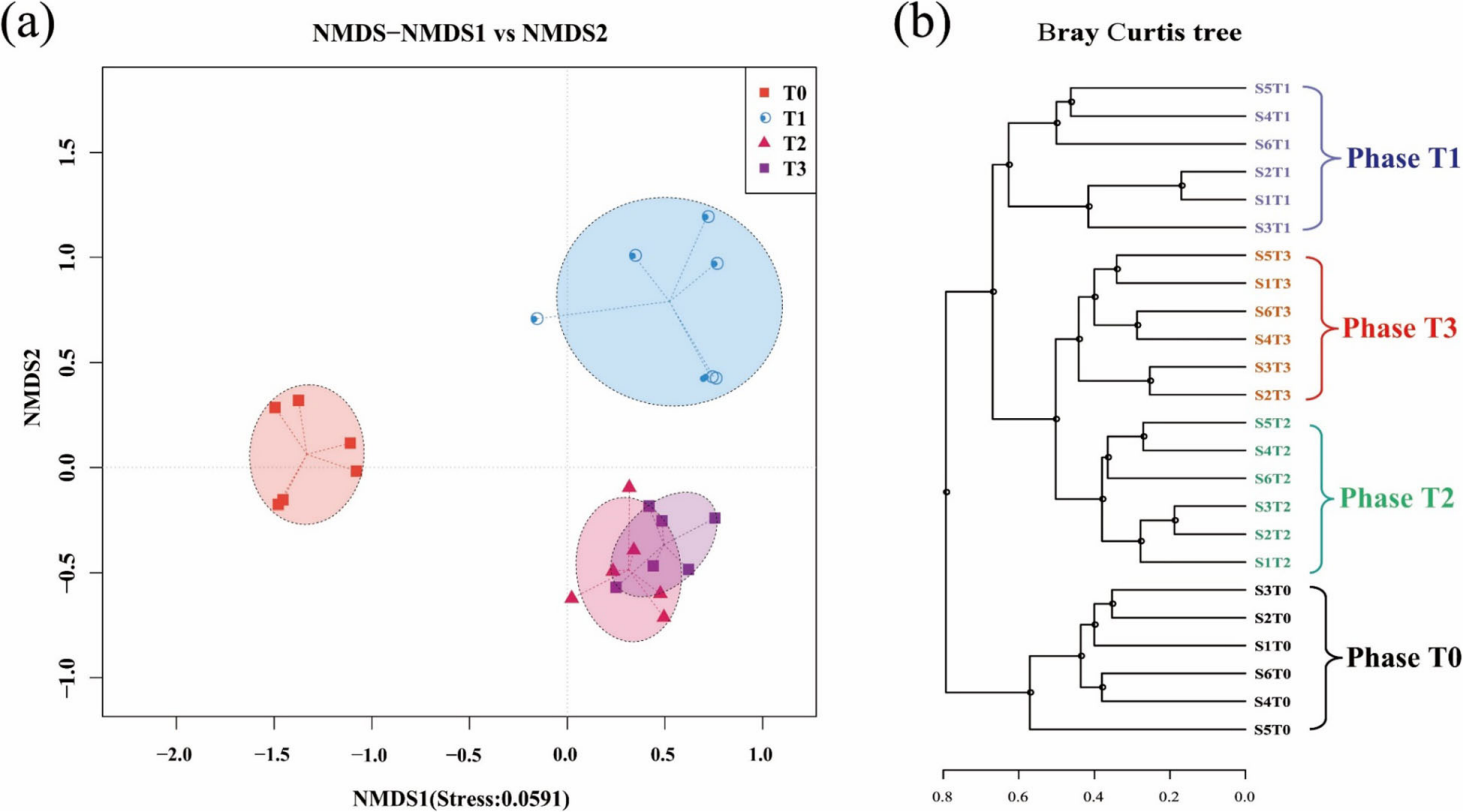
Microbial distribution differences of the collected implant samples during Peri-implantitis development were displayed with the NMDS (**a**) and UPGMA (**b**) based on Bray Curtis distance

The microbial composition was used to compare samples at different phases. The NMDS result based on Bray Cutis distance showed that the six samples at Phase T0 were clustered and were obviously different from other peri-implantitis samples. Phase T1 samples were clustered, and were different from other samples. Moreover, twelve samples at Phase T2 and Phase T3 were clustered, and were different from samples at Phase T0 and Phase T1 (Fig. 4a). The UPGMA tree based on Bray Cutis distance also showed that samples at different phases were clustered. In particular, the six samples at Phase T0 were clustered, and another eighteen samples at Phase T1 to T3 were clustered (Fig. 4b). In addition, the distance between samples at Phase T2 and Phase T3 was shorter than the distance between samples at Phase T1 and Phase T2 or Phase T1 and Phase T3. In all, the UPGMA result was in accordance with the NMDS result that the healthy state Phase T0 samples were divergent from the diseased samples at Phase T1 to Phase T3.

### Network analyses of the microbiota

**Fig. 5:**
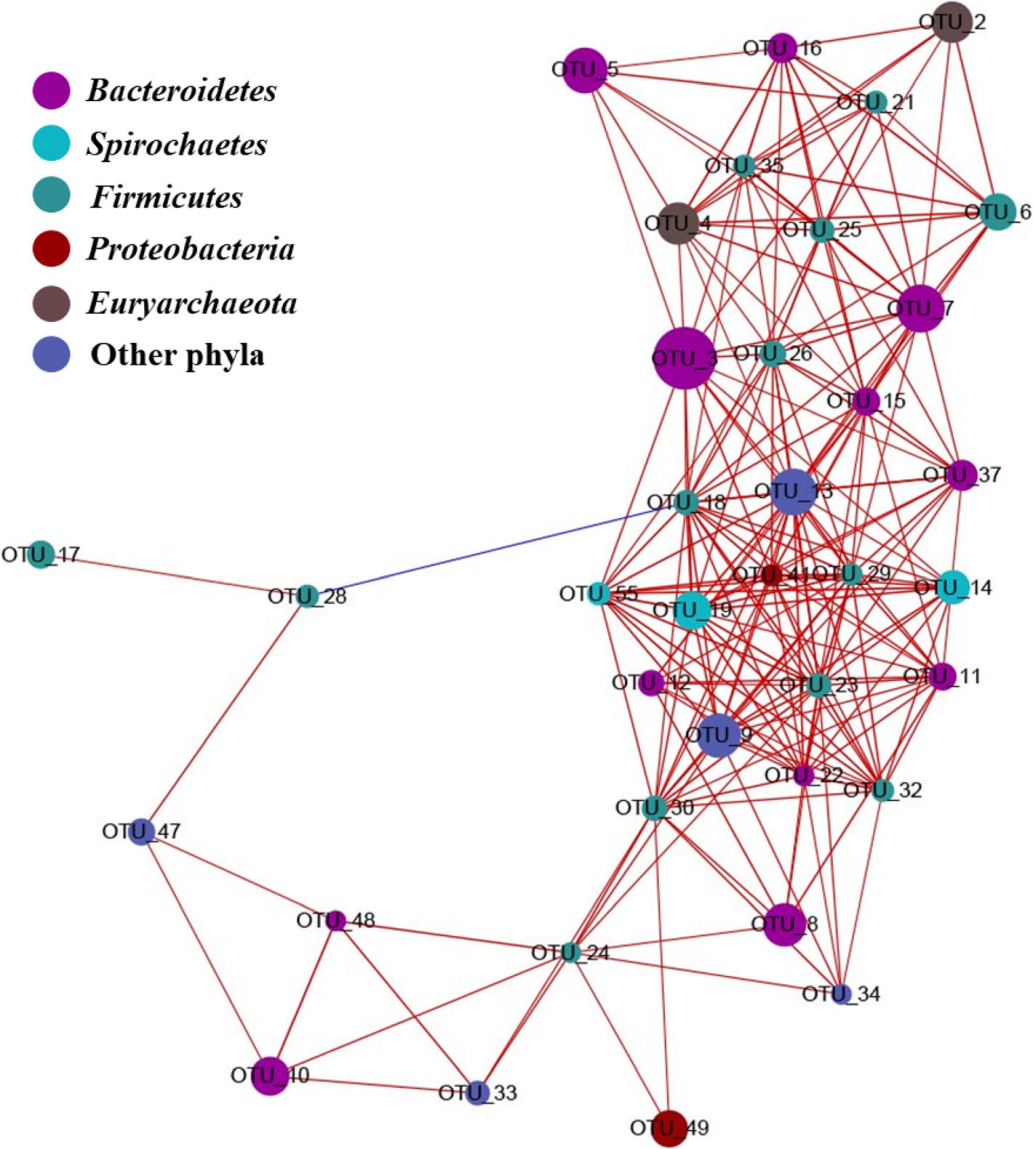
Network of co-occurring dominant OTUs (with relative composition > 0.5%) based on correlation analysis. The selection standards for strong correlation are Spearman’s *ρ* > 0.6, and significant correlation with *P* < 0.05. The size of each node is proportional to the relative abundance; the thickness of each connection between two nodes (edge) is proportional to the value of Spearman’s correlation coefficients. The line between two nodes in blue shows negative correlation between the two nodes; the line between two nodes in red shows positive correlation between the two nodes

The correlations of all the samples were calculated, and only the dominant OTUs (> 0.5%) with strong relationship (ρ > 0.6 and *P* < 0.05) were used for network analyses [31, 32]. The co-occurrence network contained 37 nodes (OTUs) and 208 edges. The compositions of these OTUs in the 24 samples were between 0.54% and 4.6% (Fig. 5). Among the 208 edges, only one edge between OTU_18 and OTU_28 indicated negative correlation. The mean edge per node was 11, and the eccentricity of the node was 3.6. The average shortest path length was 2.1 with a closeness centrality of 0.5. The average radiality of the node was 0.8, and the topological coefficient was 0.5.

Among the 37 nodes (OTUs), 13 of them were assigned to *Bacteroidetes*, and 12 of them were assigned to *Firmicutes*. The composition of *Bacteroidetes* and *Firmicutes* OTUs of the 37 OTUs was 67.57%. Interestingly, *Bacteroidetes* and *Firmicutes* were the two most dominant phyla of the 24 samples, and composed 68.89% of the microbiota (Fig. 3a). All these 37 OTUs were among the top39 OTUs of the whole microbiota. However, the most abundant OTU in the microbiota, OTU_1, was not in the network. Besides, OTU_ 122, which composed 12.34% of one sample and had an average composition in the 24 samples of 0.56%, was not in the network. Based on the connection in the network and average compositions, OTU_3, OTU_7, OTU_13, OTU_18, OTU_23, OTU_29, OTU_26, OTU_41, and OTU_30 were identified as the keystone taxa (Fig. 5). Additionally, the compositions of all these nine OTUs at Phase T0 were lower than their corresponding compositions in Phase T1 to Phase T3. Among the nine keystone taxa, four of them (OTU_18, OTU_23, OTU_26, OTU_30) were assigned to *Firmicutes*, three of them (OTU_3, OTU_7, OTU_29) were assigned to *Bacteroidetes*, OTU_13 was assigned to *Synergistetes*, and OTU_41 was assigned to *Proteobacteria*.

## Discussion

Peri-implantitis strongly affects adjacent tissues, resulting in losing the function of the osseointegrated implant [7, 8]. Peri-implantitis is caused by microbial infection, but the associated pathogenic microbes are unclear [9]. The microbial distributions between the healthy dental sites and peri-implantitis sites in subjects with chronic periodontitis had been conducted, showing diseased implants and corresponding periodontally healthy sites harbored different microbiota [33]. However, global microbial variation during peri-implantitis development was unknown. The comparison between human healthy and diseased peri-implant sites had been tried, but the human data might be affected by gender, antibiotic intake, or age [34]. The animal model can provide peri-implantitis samples with the same criteria [35]. In this study, the clinical parameters at different phases during peri-implantitis setup and progression in a canine model were collected, showing that the disease severity increased during peri-implantitis development (Fig. 2). Moreover, the microbiota changed dramatically during the shift from healthy status to peri-implantitis status.

The clinical parameters showed that the disease severity increased and the peri-implantitis model was successfully built, indicating cotton ligature around dental implant is one effective strategy for peri-implantitis induction [19]. During peri-implantitis development, microbial richness and diversity increased from the healthy phase to diseased phases (Table 1), suggesting high microbial richness and diversity were associated with severe infection. These results were different from previous results that high microbial richness was associated with healthy samples [33]. However, the increased richness and diversity in canine peri-implantitis development complements earlier study results that peri-implantitis sites exhibited higher bacterial counts compared to healthy sites [36]. The increased microbial species during in peri-implantitis development might not only derive from the original dental implant sites, but also from the oral microbiome or environmental microbiome [14]. The decreased microbial richness and diversity from Phase T2 to Phase T3 (Table 1) suggested that the host self-spontaneous progression recovery system functioned and the health status improved after removal of the ligatures.

The human oral microbiome database (HOMD) collects bacterial species distributed in the oral region and the upper respiratory tracts, and the dominant phyla were *Actinobacteria*, *Firmicutes*, and *Proteobacteria* [37]*.* Different from the microbial collection in HOMD, the main phyla in the healthy implants were *Firmicutes* (51.9%), *Bacteroidetes* (18.5%), *Fusobacteria* (11.1%), and *Proteobacteria* (7.4%), while the dominant phyla in peri-implantitis were *Firmicutes* (30.6%), *Bacteroidetes* (40.3%), *Fusobacteria* (13.9%), and *Proteobacteria* (5.6%) [15]. The decrease of *Firmicutes* and increase of *Bacteroidetes* in human peri-implantitis were the same as the microbiota variation during peri-implantitis development in this study [34], showing that phyla variation might be a common phenomenon during peri-implantitis development in human and animals. The *Synergistetes* and *Spirochaetes* compositions of human peri-implantitis sites were higher than that of human healthy sites [34, 36], and their compositions increased during peri-implantitis development (Fig. 3). Compared with healthy dental implant sites, *T. denticola* were often detected in peri-implantitis sites [36]. In this study, more *T. denticola* were detected at Phase T2 and T3 (Table 2). The results suggested a canine model can imitate human peri-implantitis, which might be useful in future human peri-implantitis therapeutic studies.

Peri-implantitis has been successfully induced by oral infection with *P*. *gingivalis* in mice, and *P*. *gingivalis* has often been identified at peri-implantitis sites [12, 38, 39]. OTU_3 and OTU_7 assigned to *Porphyromonas* were identified in this study, and the compositions of OTU_3 and OTU_7 were higher at the diseased phases (Phase T1 to Phase T3) than at the healthy phase (Phase T0), showing *Porphytomonas* might be associated with peri-implantitis. OTU_3 and OTU_7 were identified as keystone taxa, suggesting these two strains might be one potential peri-implantitis cause [10]. The compositions of methanogens were higher at Phase T2 and Phase T3, but they were not keystone taxa, indicating methanogens might not be an indicator of peri-implantitis development [40]. *Prevotella nigrescens* was also identified to be associated with peri-implantitis lesion [12, 41], but no *Prevotella* was identified in oral peri-implantitis microbiome in this study (Table 2). Though the microbiota between humans and animals were slightly different at species-level, establishment of animal oral models might give insights into microorganisms associated with human oral diseases in the future.

## Conclusion

In summary, we successfully established an experimental peri-implantitis animal model using ligature-induced method, and the increased severity of inflammation is consistent with the progression of the peri-implantitis disease as we expected. The investigation of the microbial variation during peri-implantitis development identified several OTUs associated with peri-implantitis. This study provides insight into the peri-implantitis microbiome and deepens our understanding of keystone taxa during peri-implantitis development.

## Methods

### Animals

The animal experiment had been reviewed and approved by the Ethics Committee for Animal Research in Shanghai 9th People’s Hospital, Shanghai Jiao Tong University. Three two-year-old male beagles weighing 10–15 kg were bought from Shanghai Jiagan Biotech Co. Ltd. and used for the peri-implantitis experiment in this study. The dogs were housed individually and fed a soft diet at the Animal Research Center of Shanghai 9th People’s Hospital, School of Medicine, Shanghai Jiao Tong University, Shanghai, China. All the dogs had fully erupted permanent dentition and good general health before the experiment. The dogs were sacrificed in enthanasia by intravenous administration of a lethal pentoharbital sodium dose of 120 mg/kg weight.

The remaining teeth were cleaned twice a week with 0.12% chlorhexidine and soft toothbrush. All surgical procedures were performed under general anesthesia by sedation with 2% xylazine hydrochloride and ketamine hydrochloride/zolazepam hydrochloride. The surgical sites were locally anesthetized using 2% lidocaine hydrochloride with 1:100,000 epinephrine. The surgical site was shaved and cleaned with chlorhexidine ethanol solution 0.5 mg/ml, and covered with a sterile surgical drape. The animals were made to fast for 12 h preoperatively, but allowed to drink water ad libitum.

### Teeth removal and implant placement

All the premolars (M1-M4) in the mandible (eight teeth of each dog) were extracted bilaterally from the three dogs using a minimally invasive approach (Supplementary Figure 1). After a healing period of 12 weeks, three implants (3.0 mm in diameter and 9.0 mm in length, Astra, Switzerland) were inserted at the extraction sites of both sides. Mechanical cleaning was performed to remove supragingival calculus around natural teeth 1-week pre-operation. All implants were left to heal in a submerged position for another 12 weeks, and the second-stage surgery was conducted to replace cover screws with healing abutments (width: 4 mm, height: 4 mm) (Phase T0) (Fig. 1). No plaque control procedures were performed after ligature placement.

### Induction of peri-implantitis and sample collection

A cotton ligature was ligated around each implant fixture below the healing abutment to allow for plaque accumulation and inflammatory infection [19, 42]. Two weeks later, a clinical and radiologic examination indicated that the peri-implant mucositis was established, and the samples were collected (Phase T1). Meanwhile, additional ligation was performed below the preexisting ligatures every 2 weeks, and the samples were collected after another 6 weeks (Phase T2), while radiography showed significant bone loss. Then the ligatures were removed, and the inflammation sites were left alone for self-spontaneous progression recovery. Four weeks later, samples were collected (Phase T3) (Fig. 1).

During sample collection, the sampling sites were dried with an air pistol, and samples of peri-implant sulcular fluid (PISF) were collected [39]. Sampling sites were exposed from moisture after supra-mucosal plaque was removed. Filter strips (2 mm × 10 mm) were inserted into the bottom of the peri-implant sulcus with a mild resistance at the buccomesial and buccodistal aspects of peri-implant sulcus for 30 s. All the collected strips were stored at − 80 °C before use.

### Clinical and radiographic examination

Clinical measurements were taken at the mesial, buccal, distal, and lingual sites of each implant using a UNC15 probe (Hu-Friedy, Chicago, IL, USA). The peri-implant probing depths (PD) and the relative clinical attachment levels (rCAL) in mm were measured. In addition, the Modified Plaque Index (mPI) was measured along the mucosal margin and recorded as presence (1) or absence (0); the bleeding on probing (BOP) was measured 15 s after probing and recorded as presence (1) or absence (0).

These measurements were performed by two examiners after a calibration exercise demonstrating 95.3% concordance within 1 mm for measurements of PD. The disease severity was indicated based on the measured clinical parameters.

Intra-oral radiographs of all the implant sites were taken using paralleling technique combined with long cone (Rinn XCP, Dentsply, Elgin, IL, USA).

### DNA extraction and sequencing

DNA were extracted from the collected strip samples using DNeasy Kit (Cat No./ID: 47014, Qiagen, Germany). DNA of each sample was diluted to proper concentration for gene amplification of 16S rRNA gene V3-V4 regions [43]. The Phanta Max Super-Fidelity DNA Polymerase (Vazyme Biotech, Nanjing, China) was used for 16S rRNA gene fragment amplification following the manufacturer’s procedure, and the amplification cycles were 27. Further sequencing was implemented by DeepLab Co., Ltd. (Shanghai, China).

### 16S rRNA gene analyses

USEARCH software with default parameters were used to analyze the fastq files [44]. The USEARCH UPARSE was used to classify the obtained clean reads into operational taxonomic unit (OTU) based on 97% identity, and UPARSE analysis pipeline was applied to remove chimeric reads [43, 45]. USEARCH was used to analyze the phylogenetic classification of the obtained 16S rRNA gene fragment sequences, and RDP training set (version v16) with a confidence threshold of 0.8 was used as the reference database [46]. In addition, USEARCH was further used for the α and β diversity analyses [47]. The unweighted pair-group method with arithmetic means (UPGMA) and non-metric multi-dimensional scaling (NMDS) based on Bray Curtis were analyzed by R software [47].

The co-occurrence network analysis strategy was carried out to explore co-occurrence of the abundant OTUs in the microbiota [48]. The correlation between OTUs was calculated with spearman’s method with pairwise distance. To identify keystone taxa, only strong correlation between OTUs’ relationship with ρ > 0.6, and significant correlation with *P* < 0.05 was kept. Finally, only OTUs with relative composition > 0.5% were used for further co-occurrence analysis and network construction using Cytoscape [32, 49].

## Supplementary information

**Additional file 1.**

## Data Availability

All the obtained data were deposited in Genbank database, and the accession numbers of the samples were SRR10695872-SRR10695895.
