# Supplementary material for: Oral microbial profile variation during canine ligature-induced peri-implantitis development
Source: BMC Microbiol. 2020 Sep 29;20:293. doi: 10.1186/s12866-020-01982-6 (PMC7526148; doi:10.1186/s12866-020-01982-6)
Supplement: Supplementary file 1 — Additional file 1. [file 12866_2020_1982_MOESM1_ESM.docx]

**Oral microbial profile variation during canine ligature-induced peri-implantitis development**

Shichong Qiao^1#^, Dongle Wu^1#^, Mengge Wang^2^, Shujiao Qian^1^, Yu Zhu^1^, Junyu Shi^1^, Yongjun Wei^2*^, Hongchang Lai^1*^

^1^Department of Implant Dentistry, Shanghai Ninth People’s Hospital, College of Stomatology, Shanghai Jiaotong University School of Medicine, National Clinical Research Center for Oral Diseases; Shanghai Key Laboratory of Stomatology & Shanghai Research Institute of Stomatology; Shanghai, 200011, PR China

^2^School of Pharmaceutical Sciences, Key Laboratory of State Ministry of Education, Key Laboratory of Henan Province for Drug Quality Control and Evaluation, Collaborative Innovation Center of New Drug Research and Safety Evaluation, Zhengzhou University, 100 Kexue Avenue, Zhengzhou, Henan 450001, PR China

*Corresponding authors:

Yongjun Wei

Email: [yongjunwei@zzu.edu.cn](mailto:yongjunwei@zzu.edu.cn)

Hongchang Lai

Email: lhc9@hotmail.com

| 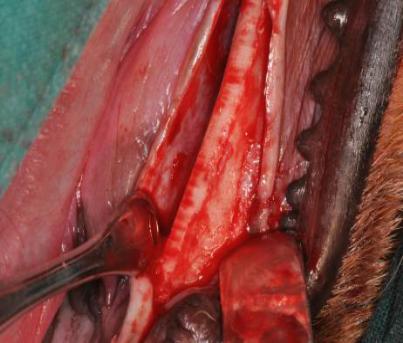  (a)  (b) | 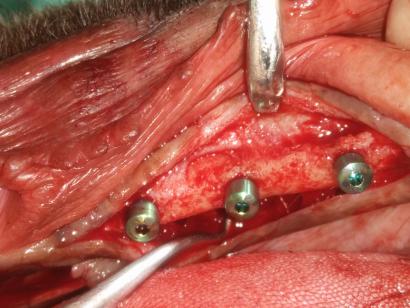 | 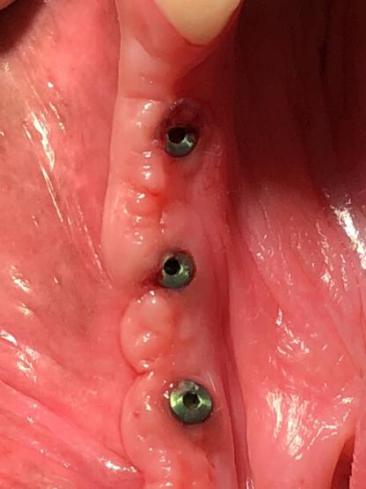  (c) |
| --- | --- | --- |
| 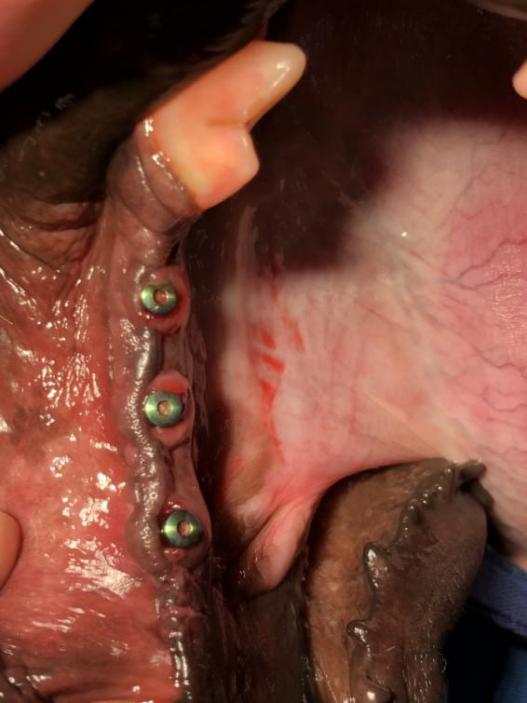  (d) | 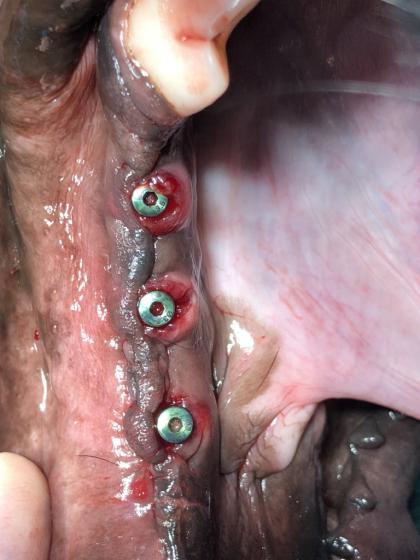  (e) | 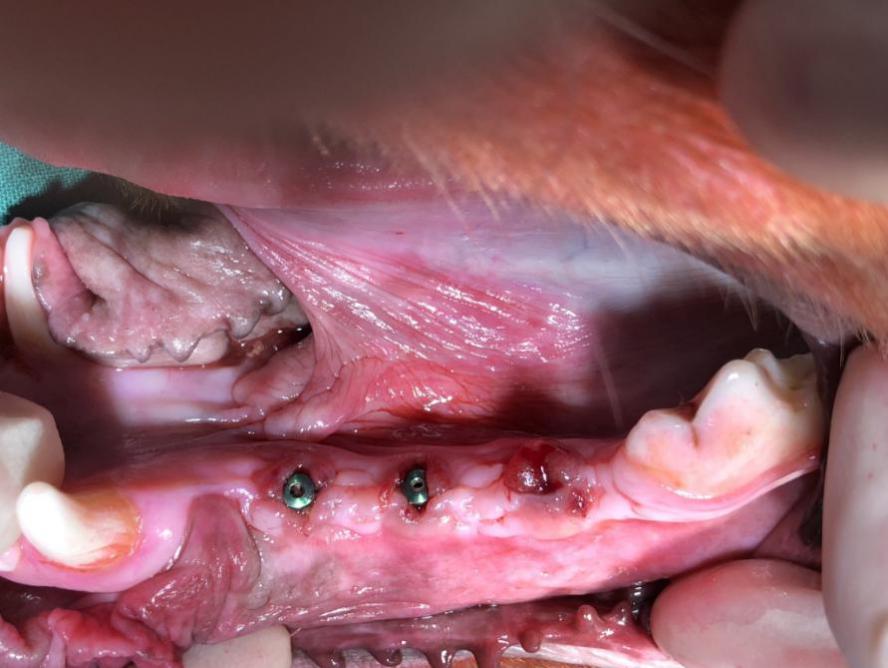  (f) |

Supplementary Figure 1 Clinical photographs during the petri-implant process and infection process. (a) Implant placement; (b)Second stage surgery; (c)ligature placement; (d) Mucositis; (e)Peri-implantitis; (f)End of spontaneous progression.


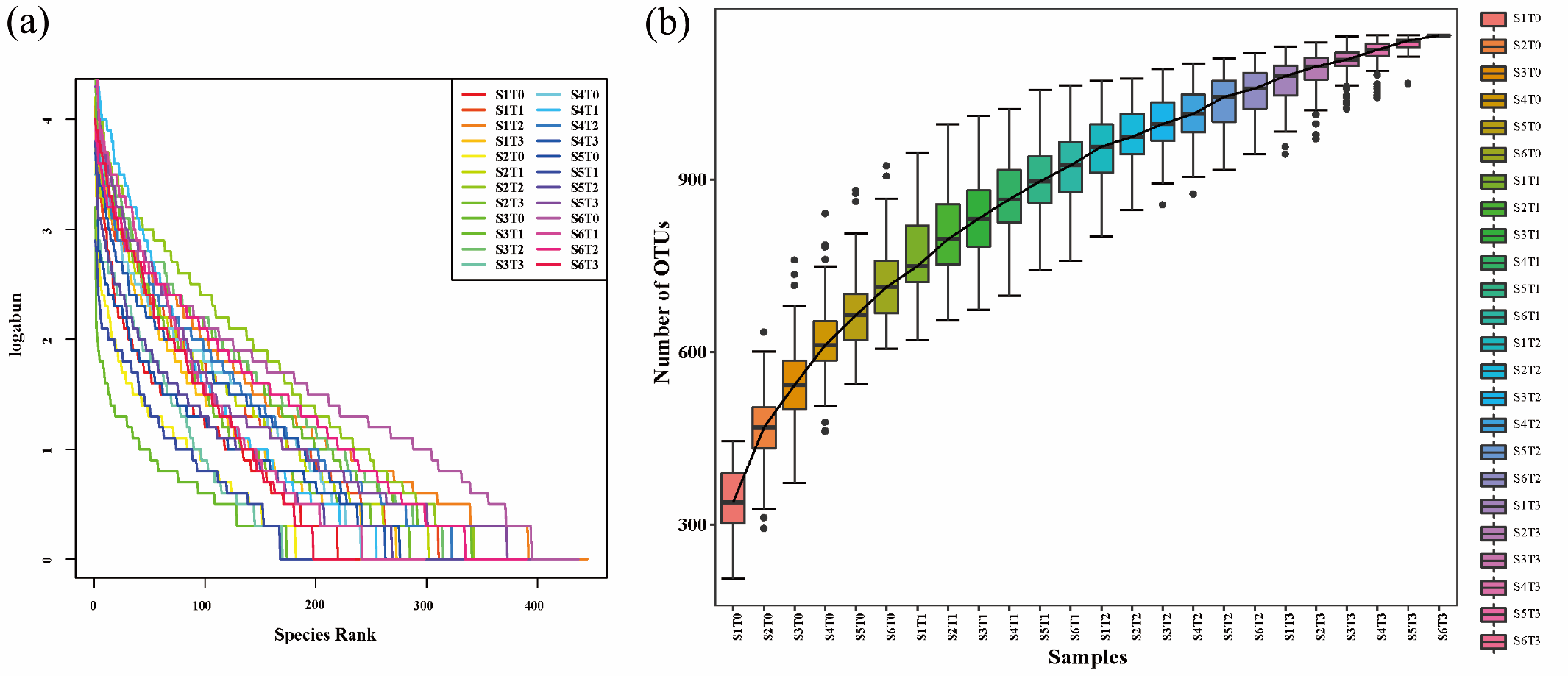


Supplementary Figure 2 Species rank and specaccum curve of the collected dog teeth samples of Phase T0 to T3.


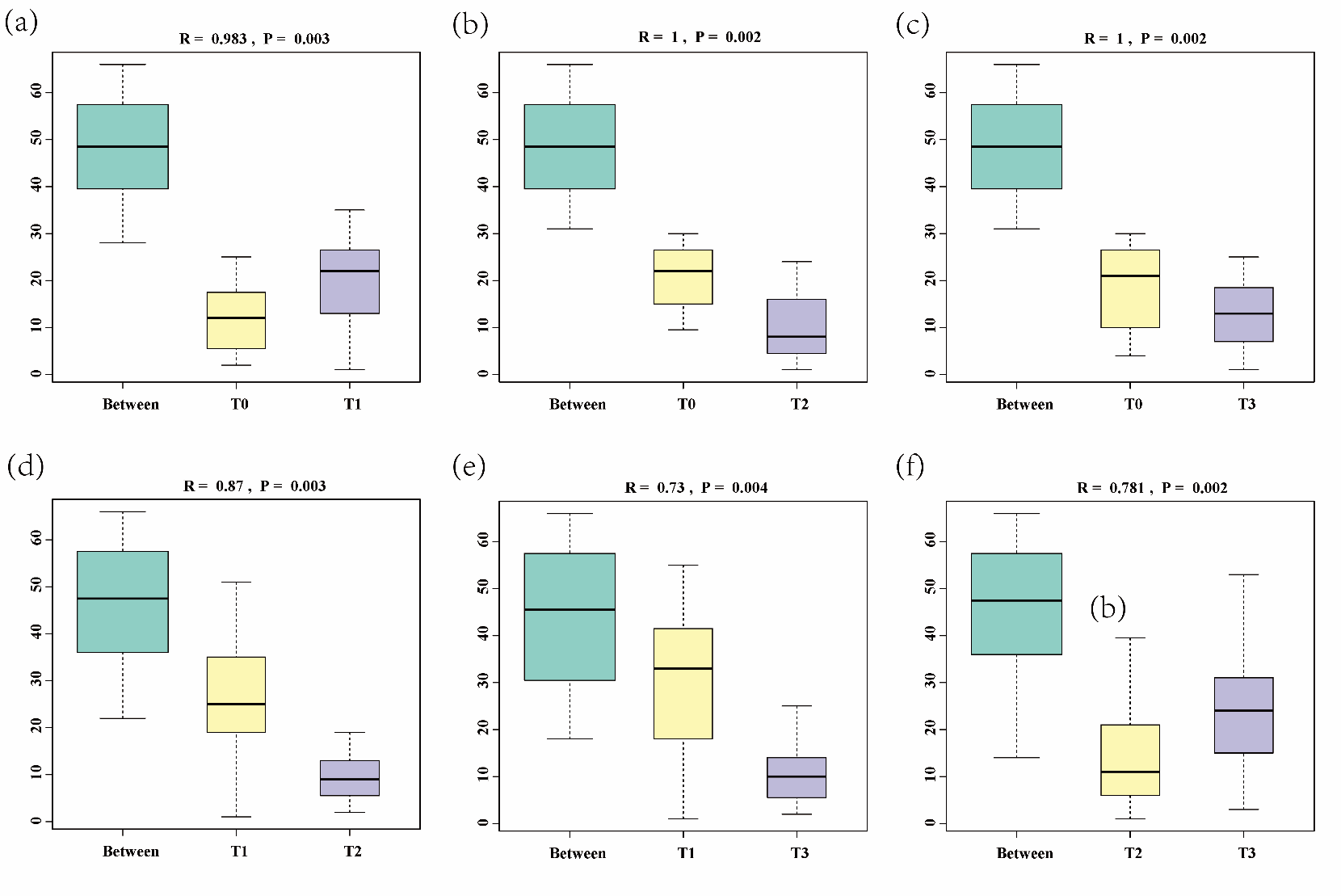


**Supplementary Figure 3** The microbial distribution differences between each two phases. (a)-(f) show the microbial distribution differences between each two phases. The 0.75 < R < 1 means highly different between the compared two groups and the group inside. The 0.5 < R < 0.75 means different between the compared two groups and the group inside. The 0.25 < R < 0.5 means different with some overlap between the compared two groups and the group inside. P < 0.01 means significant difference between the compared two groups.

Supplementary Table 1 Difference comparison of alpha diversity parameters of the four phases based on student’s t.test.

|  | Richness | Chao1 | Shannon_2 | Simpson | Dominance | Equitability |
| --- | --- | --- | --- | --- | --- | --- |
| T0 VS T1 | 0.358 | 0.349 | 0.007 | 0.003 | 0.003 | 0.017 |
| T0 VS T2 | 0.021 | 0.021 | 0.001 | 0.001 | 0.001 | 0.003 |
| T0 VS T3 | 0.806 | 0.806 | 0.011 | 0.002 | 0.002 | 0.019 |
| T1 VS T2 | 0.154 | 0.156 | 0.242 | 0.161 | 0.162 | 0.575 |
| T1 VS T3 | 0.497 | 0.483 | 0.545 | 0.557 | 0.543 | 0.811 |
| T2 VS T3 | 0.037 | 0.036 | 0.054 | 0.319 | 0.333 | 0.355 |

The yellow marked cells indicate the compared two groups display significant differences (p<0.01).

The green marked cells indicate the compared display significant differences (p<0.05).

Supplementary Table 2 Difference comparison of the four phases at the phylum-level based on student’s t.test.

|  | T0 VS T1 | T0 VS T2 | T0 VS T3 | T1 VS T2 | T1 VS T3 | T2 VS T3 | Average composition |
| --- | --- | --- | --- | --- | --- | --- | --- |
| *Firmicutes* | 0.000 | 0.000 | 0.000 | 0.015 | 0.562 | 0.023 | 41.60% |
| *Bacteroidetes* | 0.000 | 0.000 | 0.000 | 0.304 | 0.734 | 0.373 | 27.28% |
| *Spirochaetes* | 0.004 | 0.454 | 0.005 | 0.005 | 0.432 | 0.007 | 6.67% |
| *Euryarchaeota* | 0.448 | 0.007 | 0.009 | 0.005 | 0.002 | 0.079 | 5.37% |
| *Acidobacteria* | 0.567 | 0.529 | 0.770 | 0.798 | 0.490 | 0.413 | 4.64% |
| *Proteobacteria* | 0.867 | 0.031 | 0.080 | 0.049 | 0.098 | 0.655 | 4.58% |
| *Synergistetes* | 0.004 | 0.000 | 0.000 | 0.131 | 0.010 | 0.161 | 3.66% |
| *Fusobacteria* | 0.038 | 0.007 | 0.058 | 0.054 | 0.108 | 0.210 | 3.19% |

The yellow marked cells indicate the genus of the compared two groups display significant differences (p<0.01).

The green marked cells indicate two groups of the compared display significant differences (p<0.05).

Supplementary Table 3 Difference comparison of the four phases at the genus-level based on student’s t.test.

|  | T0 VS T1 | T0 VS T2 | T0 VS T3 | T1 VS T2 | T1 VS T3 | T2 VS T3 | Average composition |
| --- | --- | --- | --- | --- | --- | --- | --- |
| Above_genus | 0.00039035 | 1.8078E-05 | 0.004824358 | 0.000895644 | 0.616817756 | 0.00188107 | 27.74% |
| *Peptostreptococcus* | 0.00010365 | 9.7187E-05 | 7.55856E-05 | 0.244603667 | 0.182433283 | 0.86008879 | 17.58% |
| *Porphyromonas* | 7.1679E-05 | 0.0006199 | 0.001122247 | 0.011931466 | 0.314514073 | 0.13896509 | 15.26% |
| *Treponema* | 0.0018101 | 0.00736539 | 0.003488703 | 0.00427945 | 0.422781147 | 0.00617109 | 6.29% |
| *Bacteroides* | 0.15947459 | 0.42938915 | 0.532890159 | 0.01096635 | 0.046740434 | 0.13404797 | 3.57% |
| *Fretibacterium* | 0.00231078 | 0.00014211 | 3.32162E-05 | 0.210277247 | 0.021000036 | 0.16256754 | 3.47% |
| *Fusobacterium* | 0.03466632 | 0.00090167 | 0.043573393 | 0.052627693 | 0.093327184 | 0.24222171 | 3.02% |
| *Methanobrevibacter* | 0.4064897 | 0.00154811 | 0.000657466 | 0.00122281 | 0.000456004 | 0.46103265 | 2.64% |
| *Actinomyces* | 0.02256283 | 0.01736223 | 0.008666895 | 0.59201105 | 0.024137196 | 0.00308733 | 2.37% |
| *Streptococcus* | 0.06222163 | 0.05995138 | 0.051720479 | 0.841376685 | 0.208420621 | 0.02543167 | 1.36% |
| *Staphylococcus* | 0.02892138 | 0.02913705 | 0.030277085 | 0.457754728 | 0.297295241 | 0.38376672 | 1.26% |
| Other_genus | 0.00469836 | 0.0045912 | 0.000547745 | 0.952573553 | 0.018812025 | 0.01209017 | 16.30% |

The yellow marked cells indicate the phylum of the compared two groups display significant differences (p<0.01).

The green marked cells indicate the phylum of the compared two groups display significant differences (p<0.05).

Supplementary Table 4 Difference comparison of the four phases at the OTU-level based on student’s t.test.

|  | T0 VS T1 | T0 VS T2 | T0 VS T3 | T1 VS T2 | T1 VS T3 | T2 VS T3 |
| --- | --- | --- | --- | --- | --- | --- |
| OTU_1 | 0.000104 | 9.69274E-05 | 7.5576E-05 | 0.244897 | 0.182424 | 0.858144 |
| OTU_3 | 0.133197 | 0.000204376 | 0.000849848 | 0.183223 | 0.30861 | 0.001425 |
| OTU_7 | 0.307012 | 0.020170114 | 0.007179386 | 0.029444 | 0.010494 | 0.65062 |
| OTU_13 | 0.002164 | 0.00019407 | 4.86758E-05 | 0.277101 | 0.079069 | 0.434031 |
| OTU_5 | 0.133907 | 0.521598518 | 0.473181792 | 0.001085 | 0.020692 | 0.111458 |
| OTU_9 | 0.040296 | 0.000300906 | 0.034253963 | 0.057439 | 0.114498 | 0.151511 |
| OTU_8 | 0.081725 | 0.028518305 | 0.100922343 | 0.255774 | 0.109743 | 0.096642 |
| OTU_4 | 0.411676 | 0.001536281 | 0.000641359 | 0.001232 | 0.000457 | 0.468823 |
| OTU_2 | 0.423734 | 0.010496921 | 0.264880734 | 0.009382 | 0.212297 | 0.036152 |
| OTU_19 | 0.003141 | 0.005753649 | 0.002979372 | 0.014473 | 0.332757 | 0.008268 |
| OTU_10 | 0.099203 | 0.16488411 | 0.002549231 | 0.162434 | 0.065645 | 0.015724 |
| OTU_6 | 0.288219 | 0.002188243 | 0.106414097 | 0.001512 | 0.075479 | 0.126172 |
| OTU_14 | 0.004522 | 0.878532371 | 0.031654537 | 0.00456 | 0.409691 | 0.031835 |
| OTU_37 | 0.452737 | 0.021934745 | 0.099576073 | 0.045526 | 0.261448 | 0.230408 |
| OTU_16 | 0.256176 | 2.3724E-05 | 0.056708492 | 2.19E-05 | 0.05254 | 0.056277 |
| OTU_17 | 0.028935 | 0.029140614 | 0.030290622 | 0.482904 | 0.297399 | 0.379923 |
| OTU_15 | 0.185861 | 0.001259799 | 0.122636162 | 0.003483 | 0.41182 | 0.016537 |
| OTU_11 | 0.07858 | 0.00033914 | 0.012259917 | 0.354864 | 0.333857 | 0.838612 |
| OTU_47 | 0.10973 | 0.094405443 | 0.070513348 | 0.393817 | 0.01715 | 0.000273 |
| OTU_26 | 0.011555 | 9.55273E-05 | 0.000762824 | 0.08223 | 0.093833 | 0.806597 |
| OTU_12 | 0.0731 | 0.000590375 | 0.042762273 | 0.721208 | 0.507567 | 0.535403 |
| OTU_30 | 1.97E-05 | 0.000974816 | 0.090122506 | 0.007151 | 0.000728 | 0.101196 |

The yellow marked cells indicate the phylum of the compared two groups display significant differences (p<0.01).

The green marked cells indicate the phylum of the compared two groups display significant differences (p<0.05).
